# Defects in polynucleotide phosphorylase impairs virulence in *Escherichia coli* O157:H7

**DOI:** 10.3389/fmicb.2015.00806

**Published:** 2015-08-17

**Authors:** Jia Hu, Mei-Jun Zhu

**Affiliations:** ^1^School of Food Science, Washington State University, Pullman, WAUSA; ^2^Department of Animal Science, University of Wyoming, Laramie, WYUSA

**Keywords:** *E. coli* O157:H7, PNPase, Shiga toxin 2, prophage, Type three secretion system, intestine, epithelium, adhesion

## Abstract

Polynucleotide phosphorylase (PNPase) is reported to regulate virulence in *Salmonella, Yersinia* sp. and *Campylobacter jejuni*, yet its role in *Escherichia coli* O157:H7 has not been investigated. To gain insights into its roles in *E. coli* O157:H7 virulence, *pnp* deletion mutants were generated and the major virulence factors were compared to their parental wild type strains. Deletion of *pnp* in *E. coli* O157:H7 dramatically decreased *stx2* mRNA expression and Stx2 protein production, and impaired lambdoid prophage activation in *E. coli* O157:H7. Quantitative PCR further confirmed that the Stx2 phage lytic growth was repressed by *pnp* deletion. Consistent with reduced Stx2 production and Stx2 phage activation, the transcriptional levels of genes involved in phage lysis and replication were down-regulated. In addition, disruption of *pnp* in *E. coli* O157:H7 decreased its adhesion to intestinal epithelial cells as well as cattle colonic explant tissues. On the other hand, PNPase inactivation in *E. coli* O157:H7 enhanced Tir protein content and the transcription of type three secretion system components, including genes encoding intimin, Tir, and EspB as well as locus of enterocyte and effacement positive regulator, Ler. Collectively, data indicate that PNPase has pleiotropic effects on the virulence of *E. coli* O157:H7.

## Introduction

Shiga toxin (Stx) producing *Escherichia coli* O157:H7 is a major food safety threat that results in significant economic losses, especially in the beef industry. Stx is the major virulence factor in *E. coli* O157:H7, which causes bloody diarrhea and life threatening hemolytic-uremic syndrome (Mainil and Daube, 2005). The mortality associated with *E. coli* O157:H7 infection is due to the production and release of Stx, which is composed of a single 32-kDa A subunit and five 7.7-kDa B subunits (Paton and Paton, 1998). Stx binds to receptors on cell surface and is internalized through endocytosis, which inhibits protein synthesis and causes complications associated with *E. coli* O157:H7 infection (Johannes and Romer, 2010). In addition, *E. coli* O157:H7 has a chromosomal pathogenicity island, i.e., locus of enterocyte and effacement (LEE; McDaniel et al., 1995), which mediates intimate contact to epithelial cells, further leading to attaching and effacing (AE) lesions (McDaniel et al., 1995). The LEE is composed of five major operons (LEE1-5) that encode type three secretion system (T3SS) apparatus and effector proteins (Deng et al., 2004).

There is a plethora of studies on the regulation of LEE in *E. coli* O157:H7, which is regulated by multiple proteins such as Ler, H-NS, GrlA, CsrA through direct or indirect interaction (Bhatt et al., 2011). Compared with LEE, the regulation of Stx 2 production in *E. coli* O157:H7 has only been sparsely studied. Gene *stx2* is localized on Stx2 prophage, a lambdoid like bacteriophage, DNA that inserted to *E. coli* O157:H7 genome (Herold et al., 2004; Allison, 2007; Los et al., 2011). Stx production is generally linked to the expression of induced Stx-phage’s late genes, which results in phage lytic cycle induction and release of Stx (Wagner et al., 2001). The life cycle of prophages is controlled by phage repressor CI proteins in lambdoid phage (Ptashne and Hopkins, 1968). Mutations in the *c*I gene make a non-cleavable repressor and an uninducible 933W Stx2 prophage (Tyler et al., 2013). RecA can stimulate the self-cleavage of CI further allowing initiation of transcription from the early *P*_L_ and *P*_R_ promoters (Little, 2005). The transcription from *P*_L_ results in expression of *N* protein, which modified RNA polymerase initiating at *P*_L_ and *P*_R_ to form resistant to downstream terminators (Friedman Di, 2006). The transcription from *P*_R_ results in Q expression, which initiated the transcription from *P*_R_′ and the downstream genes expression including *stx A* and *B* (Karch et al., 1999). SOS response due to DNA damage and replication arrest can enhance *recA* expression and further stimulate Stx production (Kimmitt et al., 2000). There is also a RecA independent lambdoid prophage activation pathway for Stx production in *E. coli* O157:H7 (Imamovic and Muniesa, 2012), which is through regulation of RcsA (Rozanov et al., 1998). However, the mechanism involved in RecA-independent Stx2 933W prophage induction remains unknown (Imamovic and Muniesa, 2012). Stx prophage is also regulated by poly (A) polymerase I (PAP I), and PAP I-deficient cells showed a significant impairment of lysogenization and lytic development by Stx phages (Nowicki et al., 2015).

Polynucleotide phosphorylase (PNPase) has both 3′-5′exoribonuclease activity and 3′ terminal oligonucleotide polymerase activity, which is involved in mRNA degradation and small RNA turnover (Carpousis et al., 1999; Arraiano et al., 2010) and also serves as PAP II (Kushner, 2015). PNPase regulates T3SS expression in *Yersinia* sp. and *Salmonella* Typhimurium (Ygberg et al., 2006; Rosenzweig et al., 2007); acts as a global regulator of virulence genes in *Salmonella*, which repressed invasion and intracellular replication of *Salmonella* (Clements et al., 2002), but was required for *S.* Typhimurium gut colonization in swine (Bearson et al., 2013). It enhanced *Campylobacter jejuni* motility and its chicken gut colonization (Haddad et al., 2012). However, roles of PNPase in the colonization and virulence of *E. coli* O157:H7 had not been explored, which were examined in the current study.

## Materials and Methods

### Cell Line, Media, Bacterial Strains, and Plasmids

The human colonic epithelial cell line HT-29 was obtained from the American Type Culture Collection (ATCC^®^ HTB-38^TM^, Manassas, VA, USA). HT-29 cells were routinely cultured in Dulbecco’s Modified Eagle’s medium (DMEM; Sigma, St. Louis, MO, USA) supplemented with 10% fetal bovine serum (Sigma), 100 units/ml penicillin G, and 100 μg/ml of streptomycin (Sigma).

The *E. coli* O157:H7 EDL933, Sakai, and 86-24 strains were obtained from the STEC center at Michigan State University. *E. coli* O157:H7 was routinely grown in LB broth at 37°C with aeration. *E. coli* O157:H7 *pnp* deletion mutant was generated per the published method (Datsenko and Wanner, 2000). Briefly, using the forward primer GGCTTTACCCACATAGAGCTGGGTTAGGGTTGTCATTAGTCGCGAGGATGattccggggatccgtcgacc, and the reverse primer: CCGCCGCAGCGGAYGGCAAATGGCAACCTTACTCGCCCTGTTCAGCAGC tgtaggctggagctgcttcg, Kanamycin resistance cassette (lowercase letter) with *pnp* homology flanking sequence (uppercase letters) was PCR-amplified from pKD13 and electroporated into *E. coli* O157:H7 containing the -Red recombinase plasmid. The deletion of *pnp* was confirmed by PCR amplification using primers external to the disrupted gene (forward primer: TGTCATTAGTCGCGAGGATG; and reverse primer: GCGGAYGGCAAATGGCAACC). To construct the *pnp* complementary plasmid, the *pnp* gene was PCR-amplified from *E. coli* O157:H7 genomic DNA using primers flanking with *Kpn*I and *Xba*I restriction enzyme sites, respectively (underlined letters): ATGGTACCACGCAAACGACACCGGTTCT and AGTCTAGATTACTCGCCCTGTTCAGCAG, and cloned into pBBR1-MCS3 (Kovach et al., 1995), which was kindly provided by Dr. Mark Gomelsky. The resulting plasmid pBBR1MCS3::*pnp* and its control vector were electroporated into *E. coli* O157:H7 EDL933 *pnp* mutant strains, which resulted in six strains: (1) EDL933; (2) EDL933 *Δpnp*; (3) EDL933 *Δpnp* pBBR1; (4) EDL933*Δpnp* pBBR1::*pnp*; (5) 86-24 *Δpnp*; (6) Sakai *Δpnp*. Given that *pnp* and its downstream genes, *yhbM* or *nlpI* (Lipoprotein NlpI), *deaD* (ATP-dependent RNA helicase) and *mtr* (tryptophan permease) are located in one operon, to confirm that *pnp* deletion did not impact transcription of its downstream genes, we further compared the transcription of *yhbM*, the nearest downstream gene of *pnp*, between *pnp* deletion mutant and wild-type strain using forward primer: CAGTTTGAACAGTGCCGTGG and reverse primer: GATAACACCTCGCTCGCTGA.

### Immunoblotting

Supernatant components: overnight *E. coli* O157:H7 cultures were subcultured at 1:1,000 in LB broth for 14 h at 37°C under shaking at 200 rpm, when bacteria were in stationary phase. Bacterial cultures were centrifuged at 5,000 × *g* at room temperature for 5 min, and the supernatant was then filtered through 0.22 μm filter (Millipore, Bedford, MA, USA). Trichloroacetic acid was added to a final concentration of 10% to the supernatant and incubated overnight at 4°C to precipitate proteins.

Whole bacteria: overnight *E. coli* O157:H7 cultures were sub-cultured at 1:1,000 in LB broth for 14 h at 37°C under shaking at 200 rpm, when bacterial culture were used for protein extraction.

Protein extracts were separated by 10% SDS-PAGE, transferred to nitrocellulose membranes and assayed with antibodies specific to Stx2A (Toxin Technology Inc., Sarasota, FL, USA) and Tir (a generous gift from Dr. John Leong, Tufts University). Blotted membranes were visualized using ECL^TM^ Western blotting detection reagents (Amersham Bioscience, Piscataway, NJ, USA).

### Phage Spontaneous Induction and Enumeration

Phage enumeration assay was conducted according to our published method (Harris et al., 2012; Yue et al., 2012). Briefly, *E. coli* O157 cells were activated from frozen glycerol stock and grown in LB broth at 37°C with aeration for 8 h, which were sub-cultured at 1:1,000 in LB broth for 14 h at 37°C under shaking at 200 rpm when cultures were used to assess phage spontaneous induction. Activated bacterial culture was centrifuged at 5,000 × *g* at 4°C for 5 min. The resulting supernatant was serially diluted in a phage solution containing 10 mM CaCl_2_ (Sigma) and 5 mM MgSO_4_ (Sigma). Proper phage titration (200 μl) was mixed with 0.9 ml of the sensitive *E. coli* strain MG1655 culture and 5 ml of tempered top LB agar (0.7%, W/V) supplemented with 10 mM CaCl_2_ and 10 mM MgSO_4_. They were then mixed and poured immediately on top of the bottom LB agar plates (1.5%, W/V) containing 3 μg/ml chloramphenicol (Sigma). Plates were incubated at 37°C and plaque forming units (Pfu) were counted after 36 h incubation. Measurements for each strain had four independent replicates.

### Quantitative PCR (qPCR) Analysis of Stx2 Prophage

Shiga toxin2 prophage qPCR was conducted per published methods (Harris et al., 2012; Yue et al., 2012). Briefly, overnight bacterial cultures were centrifuged at 5,000 × *g* at 4°C for 5 min. The supernatant was then filtered through 0.22 μm filter (Millipore, Bedford, MA, USA) to ensure complete removal of bacteria. The filtrate was centrifuged at 35,000 × *g* at 4°C for 2 h. The resulting phage pellet was dissolved in sterile ddH_2_O and treated with DNase I (2 units/μl) at 37°C for 2 h to hydrolyze any remaining contaminated *E. coli* O157:H7 genomic DNA. The above phage preparation was boiled at 100°C for 10 min to denature DNase I and release phage DNA, and then used as a template for qPCR. PCR analysis of *stx2* gene was conducted using *stx2A* primers and SYBR Green master mix (Bio-Rad, Hercules, CA, USA). Absence of bacterial DNA in phage lysates was confirmed by the absence of *tufA* DNA as assayed by PCR. Stx2 phages are known to carry only one copy of the *stx2* gene, therefore the *stx2* gene copy number can be extrapolated to quantify Stx2 prophages.

### Adhesion of *E. coli* O157:H7 to Colonic Epithelial Cells

HT-29 epithelial cells were seeded in 24 well plates and cultured in DMEM media (Sigma) with 10 % FBS (Sigma) until ~90 % confluence. The medium was removed and each well was washed three times with PBS (pH 7.4). The cells in each well were challenged with 10^7^ CFU/well *E. coli* O157:H7. The HT-29 cells and *E. coli* O157:H7 were co-cultured at 37°C with 5% CO_2_ for 2 h. Then the HT-29 cell monolayers were washed three times with PBS, and lysed with 0.2% Triton X-100. Lysates were serially diluted, plated on LB, and bacterial colonies were counted after 18 h incubation (Xicohtencatl-Cortes et al., 2007; Torres et al., 2008).

### *E. coli* O157:H7 Adhesion to Cattle Colonic Explants

Terminal colons (5–10 cm from anus) were aseptically collected from *E. coli* O157:H7 free beef cattle slaughtered in the University of Wyoming Meat Laboratory and transferred to the Microbiology Lab within 10 min. Specimens were washed five times with 0.9% (W/V) NaCl and the mucosa was dissected from the underlying tissues using sterile dissecting scissors. The trimmed specimens were reduced to 8 mm round pieces using Miltex disposable biopsy punches (Cardinal Health, Cleveland, OH, USA), and transferred to the individual well of 24 well plates and cultured in DMEM medium with 5% FBS for 1 h. *E. coli* O157:H7 was added to these wells at 10^7^ CFU/well, and cultured at 37°C for 2 h (Xicohtencatl-Cortes et al., 2007). The attached *E. coli* O157:H7 cells were serially diluted, plated and enumerated.

### Quantitative Reverse Transcription PCR (qRT-PCR) Analyses

Total RNA was extracted from *E. coli* O157:H7 grown in LB broth using RNeasy Protect Bacteria Mini Kit (Qiagen, Valencia, CA, USA) and reverse transcribed using a QuantiTect Reverse Transcription Kit (Qiagen). cDNAs were used as a template for qRT-PCR analysis of selected genes using a CFX96^TM^ Real-Time PCR Detection System (Bio-Rad, Hercules, CA, USA). SYBR Green Master Mix (Bio-Rad, Hercules, CA, USA) was used for all qRT-PCR reactions. Primers for qRT-PCR are listed in **Tables 1** and **2**. Gene *gapA* was used as the housekeeping gene. Amplification efficiency was 0.90–0.99.

**Table 1:** Primer sets used for quantitative Reverse Transcription PCR (qRT-PCR) for Shiga toxin (Stx) and phage related genes.

Gene name	Product size	Direction	Sequence
*c*I	80 bp	Forward	TCACACGAAGACCAAAGGCA
		Reverse	TGCCATCGAGATGACCGAAG
*c*II	118 bp	Forward	ACCTGTCAACGCTTACCCAG
		Reverse	GATGCCATGCCAAAAGCACA
*c*III	101 bp	Forward	TTCTTTGGGACTCCTGGCTG
		Reverse	GGCTGCCCTTCCGAATCTTT
*cro*	75 bp	Forward	GAAGTTGCGAAGGCTTGTGG
		Reverse	AGTCTTAGGGAGGAAGCCGT
*gapA*	103 bp	Forward	CCAGGACATCGTTTCCAAC
		Reverse	GGTGGTCATCAGACCTTCG
*O*	104 bp	Forward	GATGCTGCAATTCAGAGCGG
		Reverse	TTTCTGGCTGATGGTGCGAT
*rcsA*	124 bp	Forward	CTCGACGATATCCTTGGCGA
		Reverse	CCTGACCTGCCATCCACATT
*recA*	70 bp	Forward	CTGGGAGCACAGCAGGTTGTCG
		Reverse	TGAACACGCGCTGGACCCAATC
*S*	124 bp	Forward	AGTTGCTGGACAGGGTTTCC
		Reverse	GCCTTACGCCGGTCTTCTTT
*stx1*	180 bp	Forward	ATAAATCGCCATTCGTTGACTAC
		Reverse	AGAACGCCCACTGAGATCATC
*stx2A*	133 bp	Forward	CGTCACTCACTGGTTTCATCAT
		Reverse	TCTGTATCTGCCTGAAGCGTAA

**Table 2:** Primer sets used for qRT-PCR for T3SS related genes.

Gene name	Product size	Direction	Sequence
*csrA*	102 bp	Forward	TCCTTCGGGGCATTTACGCCA
		Reverse	TCGTCGAGTTGGTGAGACCCTC
*eae*	139 bp	Forward	TCACTTTGAATGGTAAAGGCAGT
		Reverse	CAAATGGACATAGCATCAGCATA
*espB*	122 bp	Forward	TAAAGGGGCTGGTGAGATTG
		Reverse	CTGCGACATCAGCAACACTT
*gapA*	103 bp	Forward	CCAGGACATCGTTTCCAAC
		Reverse	GGTGGTCATCAGACCTTCG
*grlA*	71 bp	Forward	TAGAAAGTCCTGGAACAAC
		Reverse	AGACTGTCCCACAATACC
*hns*	150 bp	Forward	GCCGGACGCTGAGCACGTTT
		Reverse	CGCGGCTGCTGCTGAAGTTG
*ler*	71 bp	Forward	CCCGACCAGGTCTGCCCTTCT
		Reverse	GATGGACTCGCTCGCCGGAAC
*tir*	128 bp	Forward	AACGAAAGAAGCGTTCCAGA
		Reverse	CTGCTGCTTTAGCCTGCTCT

### Statistical Analysis

Data were analyzed as a randomized design using GLM (General Linear Model of Statistical Analysis System, SAS, 2000). Differences between means were determined using Student’s *t*-test followed by Duncan’s multiple test when appropriate. *P* < 0.05 was considered to be statistically significant.

## Results

### PNPase is Essential for Stx2 Production in *E. coli* O157:H7 EDL933 Strain

**FIGURE 1:**
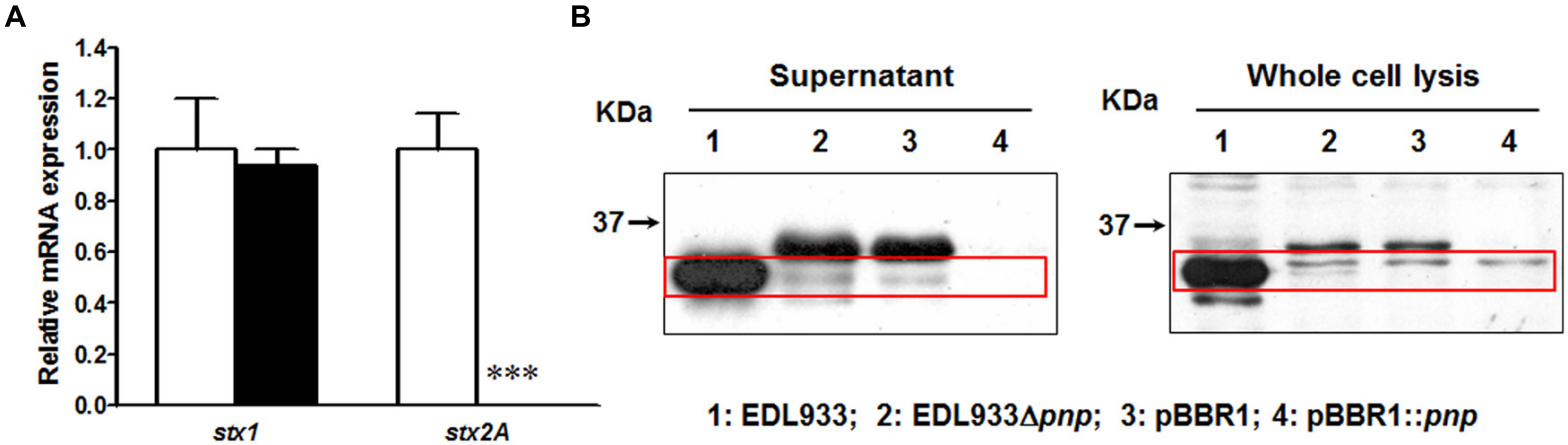
**Shiga toxin production in *E. coli* O157:H7 EDL933 strains. (A)** Relative mRNA expression; **(B)** Relative protein content. EDL933: wild-type *E. coli* O157:H7 strain; EDL933 Δ*pnp:* EDL933 *pnp* deletion mutant strain; pBBR1: EDL933 Δ*pnp* strain carrying an empty vector pBBR1; pBBR1::*pnp*: EDL933 Δ*pnp* strain complemented with pBBR1::*pnp*. □: EDL933; ■: EDL933Δ*pnp*; ***P < 0.001 (Mean ± SEM; *n* = 4).

In transcriptional level, deletion of *pnp* completely eliminated *stx2* mRNA expression in *E. coli* O157:H7 EDL933 strain without affecting *stx1* expression (**Figure 1A**). We further compared the Stx2 production. In wild-type strain, a 32 kDa band Stx2 A subunit was detected in both supernatant fraction and whole cell lysis (**Figure 1B**). Deletion of *pnp* resulted in no detection of Stx2A (**Figure 1B**). This defective in Stx2 protein production was not restored by complementation of pBBR1::*pnp* (**Figure 1B**).

### PNPase is Indispensable for Stx2 Phage Lytic Growth in *E. coli* O157:H7

**FIGURE 2:**
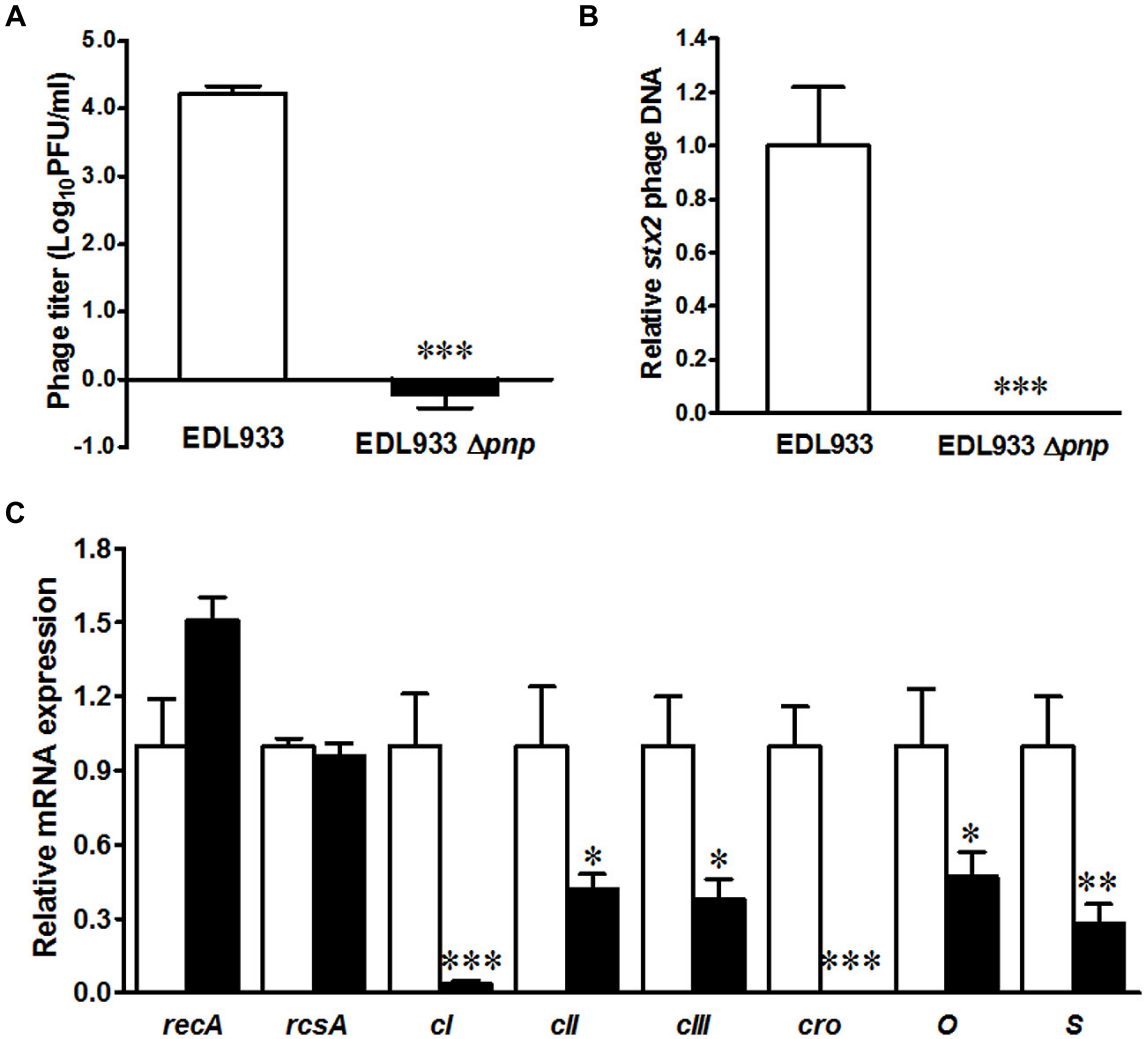
**Prophage enumeration in *E. coli* O157:H7 EDL933 wild type (□) and *pnp* deletion strains (■). (A)** Spontaneous lambdoid prophage enumeration; **(B)** Stx2 phage PCR; **(C)** Phage lysis related gene expressions. ***P < 0.001, **P < 0.01, *P < 0.05 (Mean ± SEM, *n* = 4).

Shiga toxin production is mediated by Stx2 phage activation, thus we further compared phage activation in *E. coli* O157:H7 EDL933 and its *pnp* mutant strains. Deletion of *pnp* completely abolished lambdoid phage progeny production in *E. coli* O157:H7 (**Figure 2A**). Quantitative PCR further confirmed that the *stx2* phage DNA accumulation was repressed in PNPase defective *E. coli* O157:H7 strain (**Figure 2B**). To gain insights into molecular mechanisms of PNPase mediated impairment in Stx phage activation, we further analyzed expression of phage genes regulating prophage activation. Deletion of *pnp* did not affect *recA* and *rcsA* expression (**Figure 2C**). We could not detect *cro* mRNA expressions and only detected a very low level of *c*I expression in Δ*pnp* strain (**Figure 2C**). In addition, the transcriptional levels of *c*II and *c*III, phage lysis *S* gene and phage replication gene *O* were all diminished in Δ*pnp* strain (**Figure 2C**). Altogether, these data indicated that PNPase in *E. coli* O157:H7 plays an indispensable role in Stx2 prophage lysis.

### Deletion of PNPase in *E. coli* O157:H7 Impairs Epithelial Adhesion but Enhances T3SS Encoding Transcripts and Proteins

**FIGURE 3:**
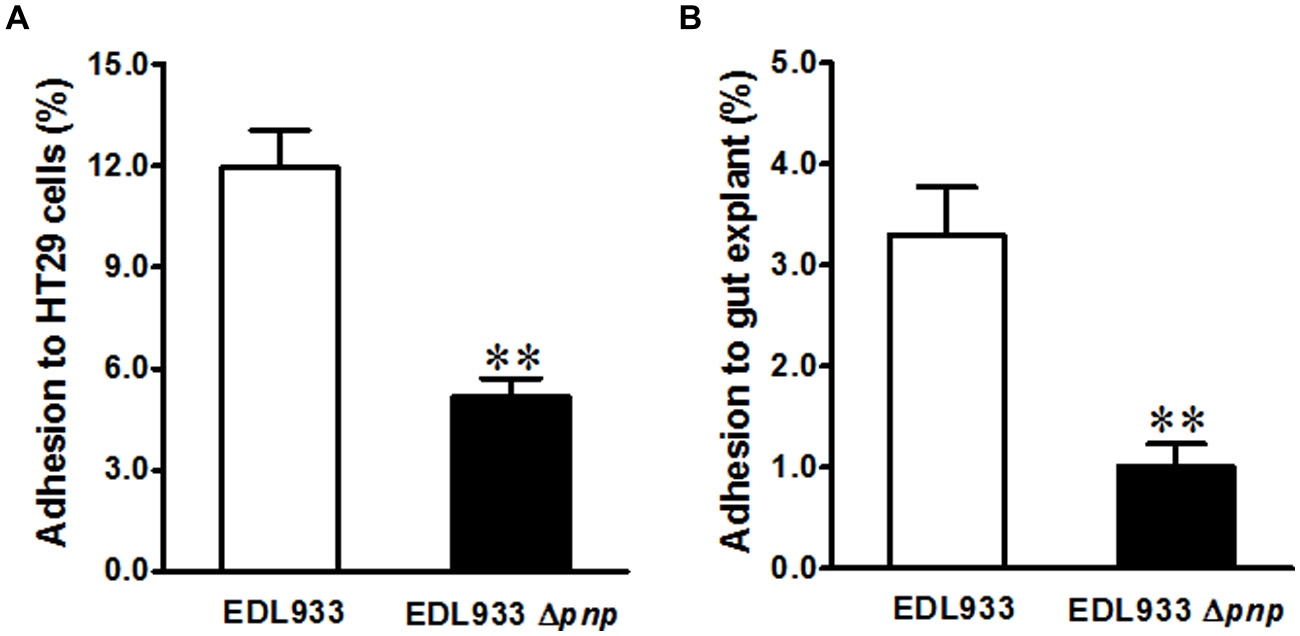
**Adhesion of *E. coli* O157:H7 to intestinal epithelium. (A)** HT-29 colonic epithelial cell line; **(B)** Cattle colonic explant tissues. EDL933: wild-type *E. coli* O157:H7 strain; EDL933 Δ*pnp:* EDL933 *pnp* deletion mutant strain, **P < 0.01 (Mean ± SEM; *n* = 8).

In addition to phage encoding Stx2 production, *E. coli* O157:H7 has a LEE chromosomal pathogenicity island that encoding T3SS apparatus and effectors, mediating intimate contact to epithelial cells (McDaniel et al., 1995). We further evaluated the significance of *pnp* deletion in *E. coli* O157: H7 epithelial adhesion. Deletion of *pnp* in *E. coli* O157: H7 EDL933 strain decreased its adhesion to colonic epithelial HT-29 cells by approximately twofold (**Figure 3A**). Consistently, adhesion to cattle colonic gut explant was reduced about fourfold in EDL933Δ*pnp* (**Figure 3B**).

**FIGURE 4:**
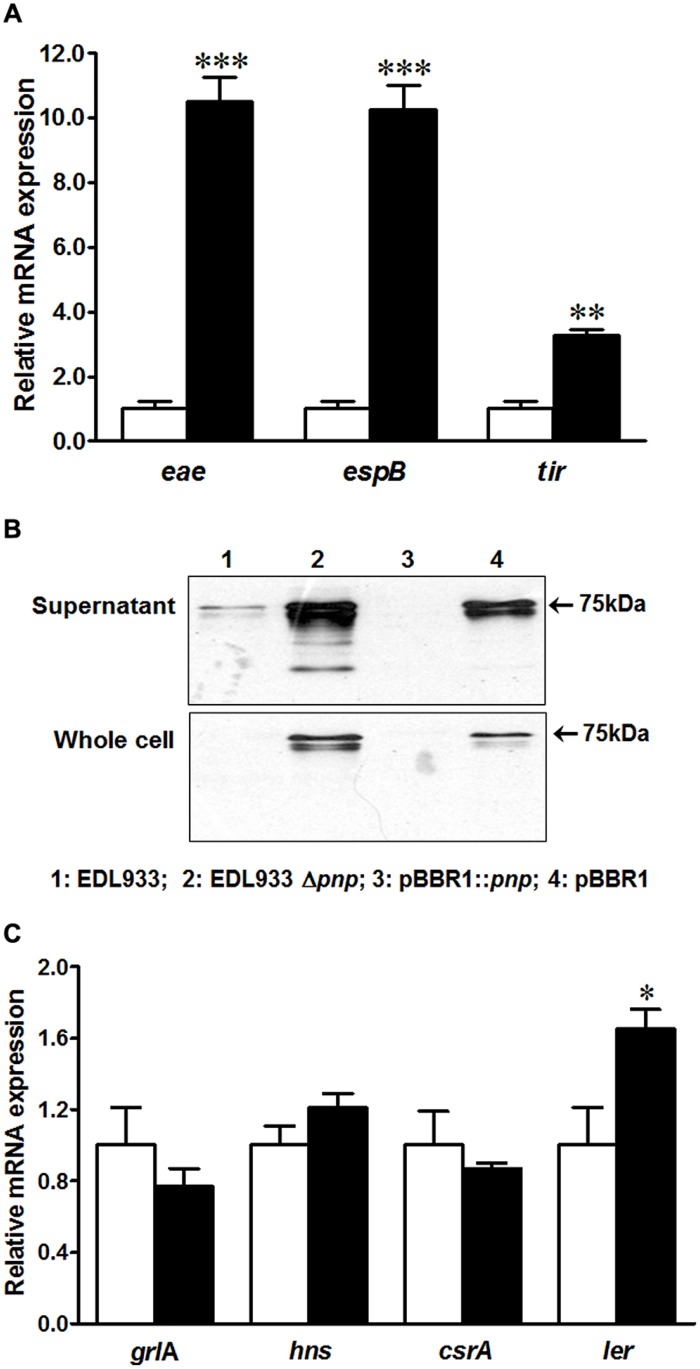
**Type three secretion system (T3SS) and its regulators in *E. coli* O157:H7 EDL933 strains. (A)** mRNA expressions of T3SS; **(B)** Tir protein content in *E. coli* O157:H7; **(C)** mRNA expression of T3SS regulators. EDL933: wild-type *E. coli* O157:H7 strain; EDL933 Δ*pnp:* EDL933 *pnp* deletion mutant strain; pBBR1: EDL933 Δ*pnp* strain carrying an empty vector pBBR1; pBBR1::*pnp*: EDL933 Δ*pnp* strain complemented with pBBR1::*pnp*. □: EDL933; ■: EDL933Δ*pnp*; ***P < 0.001, **P < 0.01, *P < 0.05 (Mean ± SEM; *n* = 4).

To understand possible factors limiting *E. coli* O157:H7 epithelial colonization in the absence of PNPase, we further analyzed T3SS apparatus and effectors transcripts and proteins in EDL933Δ*pnp* and its wild type strains. Surprisingly, the transcriptional levels of genes located on LEE region including *tir*, *eae*, and *espB* were enhanced in EDL933Δ*pnp* strain (**Figure 4A**). Tir protein production was also increased in both supernatant fraction and whole cell lysis of EDL933Δ*pnp* strain compared to the wild-type EDL933 strain (**Figure 4B**). In line with, the transcriptional level of LEE positive regulators, the *ler* transcript level, was significantly enhanced in EDL933Δ*pnp* strain (**Figure 4C**), though other regulators of LEE (*hns*, *csrA*, and *grlA*) did not differ between EDL933Δ*pnp* and its isogenic wild type strain.

### Impact of PNPase in Other *E. coli* O157:H7 Strains

**FIGURE 5:**
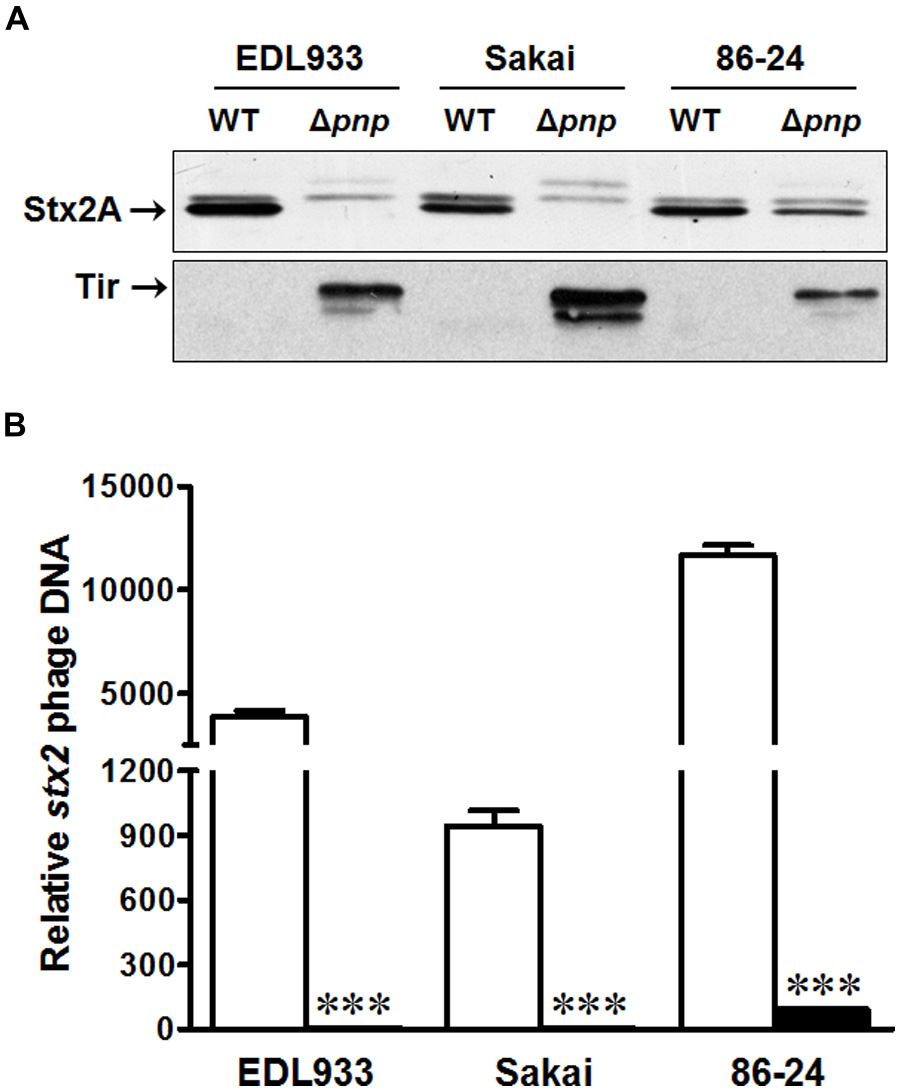
**Effects of PNPase on the virulence factors of *E. coli* O157:H7 EDL933, Sakai, and 86-24 strains. (A)** Stx 2A and Tir protein contents by immunoblotting; **(B)** *stx2* phage DNA content by qPCR. □: wild-type (WT); ■: *pnp* deletion mutant (Δ*pnp*). ***P < 0.001 (Mean ± SEM, *n* = 4).

To confirm the results from *E. coli* O157:H7 strain EDL933, we further examined the Stx2 production and Stx2 phage lytic activation in *E. coli* O157:H7 strain Sakai and 86-24. Consistent to the finding in EDL933 strain, the *pnp* deletion decreased Stx2 production (**Figure 5A**) and impaired Stx2 phage lytic growth (**Figure 5B**) in Sakai and 86-24. As in strain EDL933, Tir protein content was enhanced in both *E. coli* O157:H7 strain Sakai and 86-24 (**Figure 5A**).

## Discussion

### PNPase Regulates Stx2 Production Through Repressing Stx2 Phage Lytic Cycle

Shiga toxin is mainly responsible for the mortality associated with *E. coli* O157:H7 infection. PNPase is a bifunctional enzyme with an exoribonuclease activity and a poly (A) polymerase activity. Study herein demonstrated that PNPase plays a vital role in Stx production. The deletion of *pnp* in *E. coli* O157:H7 completely eliminated *stx2* mRNA expression and Stx2 production, which could not be recovered by complementary *pnp* overexpression. Thus we speculated that the observed effect of PNPase on Stx2 production in EDL933 strain might be due to a secondary mutation in *stx2* gene during generation of *pnp* deletion mutant. Therefore, we did three independent *pnp* deletion experiments. Different colony from three independent knock out experiments had the same Stx2 profile (data not shown). We further sequenced the promoter region of *stx2*, which revealed no alteration in the sequence of *stx2* promoter region (data not shown). Given that *pnp* and its downstream genes, *yhbM, deaD*, and *mtr* are in one operon, we questioned whether disruption of *pnp* might change the mRNA levels of *pnp* downstream genes. Thus, we further analyzed the transcription of the *pnp* nearest downstream gene, *yhbM*, and qRT-PCR analysis indicated that it was not affected by *pnp* deletion (data not shown). These testimonies showed that the decreased Stx2 production was due to *pnp* deletion. Although the polar effect of *pnp* deletion would be low in our experiment (Our experiment is in frame deletion), we can’t rule out the possibility that the polar effect of the *pnp* gene deletion cause the deficiency of Stx production.

Effective production of Stx requires phage lytic status development. Consistent with decreased Stx production, *pnp* deletion in *E. coli* O157:H7 repressed Stx2 phage lytic status, indicating that the lytic growth of 933w phage in *E. coli* O157:H7 was regulated by PNPase. CI protein is required for lysogenic establishment and has self-feedback inhibition activity, which represses prophage replication and lysis (Echols and Green, 1971). *cro* is the early lytic gene antagonizes CI and regulates phage switch from lysogenic to lytic growth during prophage induction (Svenningsen et al., 2005). Consistent with impaired Stx2 phage lytic growth, transcriptional level of *cro* were decreased in the EDL933Δ*pnp* strain. Additionally, transcriptional levels of *c*II and *c*III, phage lysis gene, *S*, and replication genes, *O*, were also repressed in Δ*pnp* compared to the wild type host. These data demonstrated that the lytic cycle of the Stx2 phage in EDL933Δ*pnp* strain was repressed, which at least partially through influencing transcripts of gene encoding proteins vital for lytic phage growth. In line with our findings, dysfunction of PAP I in *E. coli* resulted in impaired Stx phage lytic development associated with reduced transcripts of *c*II, *c*III, *cro, and O* at later time points of induction (Nowicki et al., 2015). Of note, the transcript level of *O* and *cro* are altered differently in Δ*pnp* relative to wild-type strain, which might be due to the different post-transcriptional regulation (e.g., different sRNA regulation) and warrants further research.

Similar to what observed in strain EDL933, deletion of *pnp* significantly decreased Stx2 production in Sakai and 86-24 strains. EDL933 and Sakai strains have both *stx1* and *stx2* genes, while 86-24 only has *stx2* gene. 933W prophage from EDL933 and Sakai Stx2 prophage have different sequence in early gene regulators and replication proteins (Makino et al., 1999), but almost identical sequence for structural genes (Makino et al., 1999). Compared to the genome of 86-24 Stx2 producing phage, there is a 1439 bp insertion replaced a 2091 bp sequence in 933W phage (Kudva et al., 2002). In agreement with diminished Stx2 production, Stx2 phage spontaneous lytic growth was repressed in all tested PNPase deficient strains, indicating that PNPase is important in lytic cycle development of Stx2 phage in *E. coli* O157:H7 strains.

### PNPase Reduced *E. coli* O157:H7 Intestinal Epithelial Colonization Independent of Expression Levels of T3SS

Deletion of *pnp* in *E. coli* O157:H7 decreased its adhesion to epithelial cells and colonic epithelial tissues. In agreement, *C. jejun* deficient in PNPase has reduced ability to adhere and invade to HT-29 cells, and colonize chick gut (Haddad et al., 2012). PNPase was required for *S.* Typhimurium gut colonization in swine, PNPase deficiency was associated with reduced fecal *S.* Typhimurium shedding and intestinal colonization (Bearson et al., 2013). These data collectively indicated PNPase plays a critical role in gut colonization.

*Escherichia coli* O157:H7 possess LEE pathogenicity island that encode T3SS apparatus and effector proteins, enabling its intimate contact to epithelial cells and further forming AE lesions. In contrast to decreased epithelial adhesion, we revealed that *pnp* deletion elevated the transcription level of genes located in the LEE region as well as Tir protein content, which is possibly through enhancing *ler* transcription. These data indicated that PNPase in *E. coli* O157:H7 might regulate expression and functional activity of T3SS independently, which warrants future research. The reduced T3SS functional activity as indicated by reduced epithelial adhesion might be due to the defectiveness in T3SS apparatus organization or effector secretion. Meanwhile, we speculated that the decreased intestinal adhesion might be partially due to the reduced Stx2 production. Stx regulates the distribution of nucleolin, a host cell protein that is a receptor for intimin in *E. coli* O157:H7 (Robinson et al., 2006). PNPase deletion decreased Stx2, which might disturb nucleolin distribution and further impair the adherence capacity of *E. coli* O157:H7.

Our observation was supported by previous studies in *Yersinia*, where PNPase is required for the proper T3SS function. The *pnp* mutant strain was defective in rapidly exporting T3SS effector proteins (Rosenzweig et al., 2005), less virulent in mouse, but enhanced T3SS encoding transcripts and proteins compared with its isogenic wild-type *Yersinia* strain (Rosenzweig et al., 2007). Similarly, in *S.* Typhimurium, despite of decreased swine intestinal colonization in response to PNPase deficiency (Bearson et al., 2013), T3SS-encoding transcripts and proteins were expressed at higher levels in PNPase inactivated strain (Ygberg et al., 2006). On the other hand, inactivation of PNPase in *Salmonella* increased invasion and intracellular replication as well as the establishment of persistent infection in mice (Clements et al., 2002). These implicated a complex nature of PNPases in regulating bacterial virulence.

In summary, PNPase has pleiotropic effects on the virulence of *E. coli* O157:H7. It is not only indispensable for Stx2 phage lytic cycle growth and associated Stx2 production in *E. coli* O157:H7, but also required for *E. coli* O157:H7 intestinal epithelial adhesion and regulation of type III secretion system. Alteration in PNPase activity might provide a potential strategy to reduce the virulence of *E. coli* O157:H7.

## Conflict of Interest Statement

The authors declare that the research was conducted in the absence of any commercial or financial relationships that could be construed as a potential conflict of interest.
